# Six-months follow-up of a cluster randomized trial of school-based smoking prevention education programs in Aceh, Indonesia

**DOI:** 10.1186/s12889-015-2428-4

**Published:** 2015-10-24

**Authors:** Teuku Tahlil, Richard J. Woodman, John Coveney, Paul R. Ward

**Affiliations:** Nursing Faculty, Syiah Kuala University, Banda Aceh, 23111 Indonesia; Flinders Centre for Epidemiology and Biostatistics, School of Medicine, Flinders University, Adelaide, SA 5001 Australia; Discipline of Public Health, School of Health Sciences, Flinders University, Adelaide, SA 5001 Australia

**Keywords:** School-based program, Tobacco smoking, Adolescents, Muslim context, Indonesia

## Abstract

**Background:**

Smoking prevention programs have been taught in schools to reduce the high smoking prevalence and its related problems among adolescent populations. Although short-term benefits have been observed, the long-term effectiveness of such programs appear to be inconsistent. This study aims at investigating the long-term impact of both health and Islamic focused interventions amongst students in Indonesia.

**Methods:**

At 6 months after completion of the interventions, 427 of the original 447 participants (control group = 128, intervention groups = 299) from a school-based cluster randomized control trial were re-assessed for their smoking knowledge, attitudes, intentions and behaviours using a self-report questionnaire. Data was analyzed according to the study’s 2 × 2 factorial design with adjustment for baseline scores, school and classroom clustering effects and multiple comparisons.

**Results:**

Compared to the control group, significant long term effects were found for the health-based intervention program in improved health (β = 4.3 ± 0.4, *p* < 0.001), Islamic (β = 1.1 ± 0.4, *p* = 0.01) knowledge and a reduction of smoking attitudes (β = −11.5 ± 1.8, *p* < 0.001). For the Islamic-based intervention programs there was an improvement of health (β = 3.7 ± 0.4, *p* < 0.001) and Islamic (β = 2.2 ± 0.5, *p* < 0.001) knowledge and a reduction towards smoking attitude (β = −6.0 ± 1.9, *p* < 0.01) and smoking behaviors in the past month (OR = 0.1, 95 % CI = 0.0–0.8, *p* = 0.03). The effects were greater but less than additive in the combined group for health (β = −3.2 ± 0.9, *p* < 0.001 for interaction) and Islamic knowledge (β = −2.3 ± 0.9, *p* = 0.01 for interaction) but were additive for smoking attitudes (β = 6.1 ± 3.2, *p* = 0.07 for interaction). No significant effects on smoking intentions were observed at 6 months follow-up in the health or Islamic-based intervention programs.

**Conclusion:**

School-based programs can provide long term benefits on Indonesian adolescents’ smoking knowledge and attitudes. Tailoring program intervention components with participants’ religious background might maximise program effectiveness. A larger and more encompassing study is now required to confirm the effectiveness of this new Indonesia culturally-based program. Adolescents in similar areas might also benefit from this type of school-based smoking cessation program.

**Trial registration:**

Australian New Zealand Clinical Trial Registry, ACTRN12612001070820

## Background

Much progress has been made concerning the implementation of tobacco control measures worldwide [1], but tobacco use and its associated harms remains high across the globe. Worldwide, over 1.3 billion people smoke [2], around 6 million have died due to tobacco and annually over half a trillion dollars is spent globally to cover monetary loss due tobacco-related effects [1]. Tobacco smoking causes many diseases [3] and is accounting for 8.4 % of disease burden among men and 3.7 % among women [4].

Indonesia is the world’s third largest country in tobacco consumption [5]. Approximately 29.3 % of Indonesians smoke tobacco (64.9 % of males and 2.1 % of female) [6]. Indonesians smoke about 12.3 sticks of tobacco per day [6] and spend about 6.3 % of their income on tobacco [7]. The prevalence rates of current tobacco use among adolescents are 13.5 % (24.1 % for boys, 4 % for girls) [5]. Despite the high proportion of tobacco smoking, tobacco control measures are currently very weak within Indonesia [1].

Following observed benefits from short-term school-based smoking cessation programs, there has been an increasing focus on examining the longer term effectiveness of such programs. Although numerous programs over the past 40 years have been developed [8], findings of systematic reviews provide different conclusions about the long-term effectiveness of school-based smoking prevention programs with one review of 8 randomized trials with follow-up by age 18 or grade 12, at least 1 year after program intervention completion, concluding there was insufficient long term evidence to recommend school-based smoking prevention programs [9]. Conversely, other reviews [10, 11] had suggested there was evidence for long-term effectiveness of school-based smoking prevention programs.

We have previously developed and tested three types of school-based smoking prevention programs comprising a health, Islamic, and combined health and Islamic-based program amongst adolescents in a western area of Indonesia [12]. The immediate effects of the programs which showed positive improvement in participants’ smoking knowledge, attitude, intentions, and behaviours at one week after the programs completion have been reported elsewhere [12]. The current paper assesses the program’s effectiveness at 6 months follow-up. Evaluation included a re-assessment of the participants’ smoking knowledge, attitudes, intentions, and behaviours.

## Methods

### Design and randomization

Eight junior high schools from the capital of Aceh Province, Indonesia were recruited for participation. Two classes in each school with about 15 to 16 students per class were selected and assigned to one of the three intervention arms (the health, the Islamic, or the combined program) or to a control arm. The selected schools were approved by the head of the education department in the district and randomized using random number generation procedure in Excel. There was no difference between schools in terms of schools location, students size and their background characteristics. Additional information about the school selection process, characteristics and randomization procedures can be read elsewhere [12].

All school students completed three waves of program evaluation: (1) Baseline: one week before intervention, (2) Post-intervention: within 1 week of the 8-week intervention completion (3) 6 months follow-up: 6 months after the intervention completion (8 months after baseline). Students in the intervention groups received eight sessions of smoking prevention education. Students in the control group received no education intervention.

### Participants

**Fig. 1:**
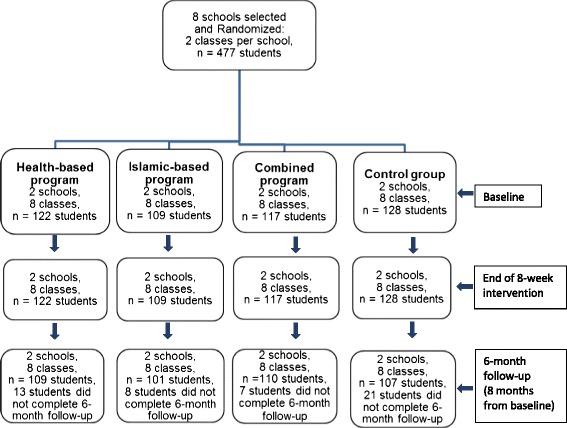
Study Participants by groups from baseline to 6 months follow-up

Study participants comprised adolescents aged between 11 and 14 years. A power calculation was conducted based on mean and changes and standard deviations of knowledge scores (as the primary outcome measure) from a pilot study. This showed that the required sample size was 480 to provide 80 % power for comparing group differences at a 2-sided alpha level of 0.05 [12]. Initially a total of 477 students participated in the study. Of the 477 students, 476 (99.8 %) completed program evaluation at baseline and following intervention and 427 (89.5 %) provided information at all 3 program evaluation time-points. The retention rates at 6 months for the individual groups were 109 (89.3 %) for the health-based program; 101 (92.7 %) for the Islamic-based program; 110 (94.0 %) for the combined program, and; 107 (83.6 %) for the control group. Reasons for dropout included absenteeism, school events, leaving or transferring to other schools. Figure 1 provides detailed proportions of the study participants by group assignment throughout the study process.

### Interventions

Program interventions were developed on the basis of systematic review of available school-based smoking prevention programs worldwide, published between 2004 and 2009. This review focused on trends in school-based smoking prevention programs, and then synthesized findings of this review into what is called the Health-Based Intervention program in this study. We also reviewed all available literature on religious based education programs, including those relating to smoking prevention and cessation, and synthesized these into what is called the Islam-based Intervention. Additionally, a qualitative research with teachers and policy makers in education from Aceh, Indonesia, was conducted in order to develop more culturally appropriate interventions and match with current curricula [13]. The programs employed both social influence and competence curricula. The health-based program provided students health-based information and skills surrounding smoking prevention including information about historical perspective of tobacco smoking and smoking behaviours in Indonesia, tobacco smoking effects, national regulation about tobacco smoking, refusal skills, assertiveness, and stress management. The Islamic-based program taught students information and relevant skills about smoking prevention from an Islamic perspective, and this included information about Islam; tobacco smoking among Islamic society; Islamic view about health, tobacco smoking, and healthy living techniques. The combined program comprised key concepts of the health- and the Islamic-based approaches including the Islamic concept, historical perspective of tobacco smoking in Indonesia, effects of tobacco smoking, Islamic and national rules on tobacco smoking, refusal skills, and healthy living techniques in Islam.

The curricula were divided into eight two-hour sessions and administered in the students usual classrooms during school hours. Program providers included school teachers and health professionals for the health-based program; Islamic leaders and school teachers with in-depth knowledge in Islamic teaching about smoking prevention for the Islamic-based program, and; the combination of school teachers, health professionals, and Islamic leaders for the combined program. The schools integrated program activities into relevant school subjects. Providers used various interactive teaching methods that were culturally appropriate to students such as group/class discussions, brain storming, role play, and storytelling. One day training and program material including teaching material and instruction were provided to providers and students in order to prevent any biases in program delivery and contents. Also, meetings and supervision were conducted during program implementation, to ensure that program implementation was carryout as planned. Further explanation about program content and delivery methods can be found elsewhere [12].

### Data collection and measures

Data were collected using a paper-based, close-ended response questionnaire. This self-report questionnaire was conducted at each of the study’s three data collection time-points. Students completed the questionnaires in their classrooms during school hours which lasted about 80 min. To ensure that students could honestly provide their responses, students were informed about the confidential nature of their responses. Also, no school teacher or administrators were involved in these tests. Research staffs with the help of external program providers, who were both not affiliated with the schools, administered the tests under the supervision of researchers. There was no school teacher and school staff in class room during the tests.

Overall, the questionnaire assessed demographic characteristics of the study participants and program outcome measures. The development process of the questionnaire has been previously explained elsewhere [12]. This reports on the development of the primary outcome measures which were an assessment of participants’ smoking knowledge, attitude, intentions, and behaviours. These comprised relevant questions from previously valid and reliable questionnaires and were complemented with questions specifically designed for assessing the Islamic-based knowledge outcomes. The instruments were pilot tested for validity, internal reliability and test-retest validity with a 2 week interval between tests.

It has been previously reported [12] that the questionnaire comprised 71 items, these included: (1) 4 items for assessing students’ demographic participants; (2) 20 items for assessing health knowledge outcome (Cronbach’s alpha was 0.88 at Test 1 and 0.90 Test 2, kappa between 0.08 and 0.53); (3) 20 items for assessing Islamic knowledge outcome (Cronbach’s alpha was 0.79 at Test 1 and 0.88 Test 2, kappa between 0.02 and 0.66); (4) 25 items for assessing smoking attitudes outcome (Cronbach’s alpha was 0.87 at Test 1 and 0.86 Test 2, kappa between 0.02 and 0.52); (5) 3 items for assessing smoking intentions outcome (Cronbach’s alpha was 0.74 at Test 1 and 0.84 Test 2, kappa between 0.35 and 0.49); (6) 3 items for assessing smoking behaviours outcome (Cronbach’s alpha was 0.88 at Test 1 and 0.80 Test 2, kappa between 0.77 and 0.79).

Demographic information included sex, age (in years), year of study (in grades), and living conditions (with both parents, one parent and stepfather/mother, one parent only, relatives, or others). The program outcome measures were focused on smoking knowledge, attitudes, intentions and behaviours. The knowledge outcome was divided into a health and an Islamic section (20 items each) and formatted as multiple choice questions. The health section measured participant’s knowledge about the negative effects of tobacco smoking, national tobacco smoking prevalence and its regulation in Indonesia. The Islamic section measured participant’s knowledge about Islamic concepts, Islamic views about health, tobacco use and regulation among Muslims.

The smoking attitude section asked participants to indicate their agreement on 40 positive/negative statements about the physical, social and economic effects of tobacco smoking and tobacco smoking policy. The items to measure this variable were rated on 5-point Likert scales, ranging from 0 (strongly disagree) to 4 (strongly agree). The smoking intentions were measured by three items assessing participant’s intention to smoke tobacco next year, during senior high school and when older. These items were rated on 5-point Likert scale, ranging from 0 (certain not to smoke) to 4 (certain to smoke). The smoking behaviours assessed the amount of tobacco consumed by a participant in the past week, month, and lifetime. Potential responses ranged from 0 (never smoked) to 4 (three to five cigarettes) for the past week’s smoking; 0 (never smoking) to 6 (more than 20 cigarettes per day) for the past month’s smoking; and 0 (never smoking) to 8 (over 100 sticks of cigarettes) for lifetime’s smoking. The majority of questions for the health-related aspects of smoking were adopted from previous studies [14–24] and matched with educational material of the current study and cultural background of the study participants. Items for the Islamic knowledge aspect were based on program materials. The questionnaire appeared to be valid and reliable [12].

### Ethical approval

The Social and Behavioural Research Ethics Committee (SBREC) of Flinders University, Australia and the Ethical Clearance Committee of Medical Faculty of Syiah Kuala University, Indonesia provided ethical approval for the study. Written consents for participation from schools’ principals, parents/guardians/significant others, and students were requested. The students were informed about the study’s purposes, procedures, potential risks and benefits, and other ethical consideration including the voluntary nature of their participation by oral and information sheet.

### Data analysis

Data analysis was performed using Stata version 12.0 (StataCorp, Texas, USA). Between groups comparisons of demographic information was assessed using chi-squared tests. Differences between groups at baseline of the outcome variables was assessed using Analysis of variance (ANOVA) for smoking knowledge and attitude, and using chi-squared tests for smoking intentions and behaviours. The main effects of Health and Islam programs and the interaction between Health x Islam at 6 months were assessed using generalized linear models with the 6 month outcome variable as the dependent variable and with indicator variables for Health and Islam, and a Health x Islam interaction term and a term for baseline scores. The degree of clustering within classrooms and schools for each outcome was assessed using mixed effects regression models with classroom and school identifiers added as random effects. Intra-class correlation coefficients (ICC) were used to report the clustering effects. An additional Analysis of Covariance (ANCOVA) with Bonferroni-adjustments for comparisons of the 3 intervention groups with the control group was conducted to aid interpretation of the study findings. Further explanation of the data analysis procedure has been described in detail elsewhere [12].

## Results

### Characteristics of participants at 6 months follow-up

Table 1 compares background characteristics of the 427 study participants at 6 months follow-up. Overall, participants were mostly girls (58.8 %) seventh graders (50.1 %), mostly aged over 12 years (92.8 %) and lived with both parents (80.8 %). There were no significant differences between the intervention groups and the control group (all *p* > 0.05).

**Table 1:** Characteristics of participants by groups at 6 months follow-up visit

Characteristics	Health (*n* = 109)	Islamic (*n* = 101)	Combined (*n* = 110)	Control (*n* = 107)	*p* values^a^
Sex					0.92
Boys (%)	42.6	42.2	38.5	41.4	
Girls (%)	57.4	57.8	61.5	58.6	
Age					
11 years (%)	1.6	1.8	2.6	0.8	0.50
12 years (%)	23.0	32.1	31.6	38.3	
13 years (%)	48.4	45.9	42.7	39.8	
14 years (%)	27.0	20.2	23.1	21.1	
School grade					
7th (%)	45.9	51.4	51.3	47.7	0.81
8th (%)	54.1	48.6	48.7	52.3	
Residence status					
With both parents (%)	88.5	89.0	77.8	80.5	0.10
With one parent and step parent (%)	0.8	0.9	6.0	2.3	
With one parent only (%)	5.7	6.4	10.3	6.3	
With relatives (%)	4.1	3.7	4.3	8.6	
Others (%)	0.8	0	1.7	2.3	

^a^using chi-squared test of association

### Program effects on knowledge at 6 months follow-up

**Fig. 2:**
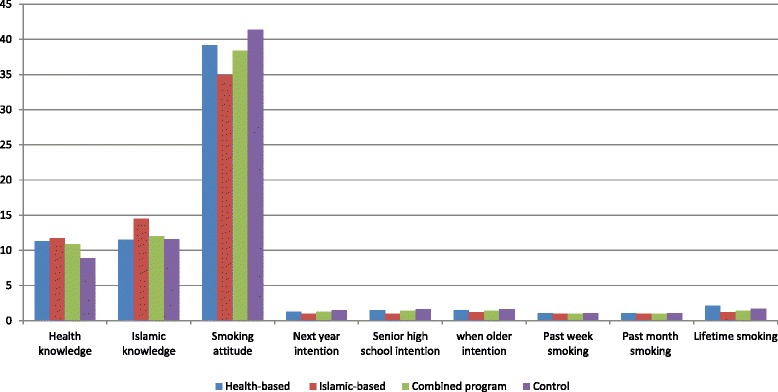
Groups comparison in knowledge, attitudes, intentions and behaviors at 6 months follow-up

The main effects and interaction effects of the health and Islamic-based intervention programs on health and Islamic knowledge at 6 months follow-up are presented in Table 2. There was a significant main effects of the health (β = 4.3 ± 0.4, *p* < 0.001) and Islamic-based intervention (β = 3.7 ± 0.4, *p* < 0.001) on health knowledge improvement at 6 months follow-up compared to the control group. Interaction effects between health and Islamic-based intervention were significant (β = −3.2 ± 0.9, *p* < 0.001), suggesting that the effect of the combined program on health knowledge depended upon inclusion of Islamic-based intervention component and vice versa, i.e. the effects were not completely additive in the combined group. The effects were relatively homogenous between participants within the same classroom (ICC = 0.10) indicating that there was an additional classroom effect in addition to an individual learning effect. Groups’ differences in health knowledge are presented in Fig. 2 and Table 3. There were significantly greater health knowledge scores after adjusting for baseline (*p* < 0.001) for all 3 intervention groups when compared to control group (Table 3).

**Table 2:** Knowledge and attitude scores, intentions and behaviors at 6 months follow-up and the baseline adjusted program effects (β) (*n* = 427)

Outcomes	Health intervention				Islamic intervention				Health x Islamic Interaction		ICC^f^
	Health (*n* = 219) (mean ± SD)	Non health (*n* = 208) (mean ± SD)	β ± SE^a^	*p* value^d^	Islamic (*n* = 211) (mean ± SD)	Non Islam (*n* = 216) (mean ± SD)	β ± SE^b^	*P* value^d^	β ± SE^c^	*p* value^e^	
Health Knowledge	11.1 ± 1.9	10.2 ± 2.6	4.3 ± 0.4	<0.001	11.3 ± 2.0	10.1 ± 2.5	3.7 ± 0.4	<0.001	−3.2 ± 0.9	<0.001	0.10
Islamic Knowledge	11.7 ± 2.7	13.0 ± 2.4	1.1 ± 0.4	0.01	13.2 ± 2.7	11.6 ± 2.3	2.2 ± 0.5	<0.001	−2.3 ± 0.9	<0.01	0.09
Attitude	38.8 ± 7.3	38.2 ± 8.6	−11.5 ± 1.8	<0.001	36.7 ± 7.1	40.3 ± 8.4	−6.0 ± 1.9	<0.01	6.1 ± 3.2	0.07	0.07
	Health intervention				Islamic intervention				Health x Islamic Interaction		ICC^f^
	Health n (%)	Non health n (%)	OR (95 % CI)^a^	*p* value^d^	Islamic n (%)	Non Islam n (%)	OR (95 % CI)^b^	*p* value^d^			
Intention to smoke next year	10 (4.6)	16 (7.7)	0.4 (0.1,1.3)	0.14	8 (3.8)	18 (8.3)	0.4 (0.1,1.6)	0.20	*		0.12
Intention to smoke in senior high school	13 (5.9)	20 (9.6)	0.4 (0.1, 1.3)	0.13	9 (4.3)	24 (11.1)	0.3 (0.1, 1.2)	0.10	*		0.12
Intention to smoke over 50	17 (7.8)	22 (10.6)	0.5 (0.2, 1.2)	0.13	15 (7.1)	24 (11.1)	0.6 (0.2, 1.4)	0.24	*		0.07
Past week smoking	3 (1.4)	4 (1.9)	0.5 (0.2,1.5)	0.24	0 (0)	7 (3.2)	0.0 (0)	**	*		0.24
Past month smoking	6 (2.7)	8 (3.9)	0.4 (0.2, 1.0)	0.04	1 (0.5)	13 (6.0)	0.1 (0.0, 0.8)	0.03	*		0.10
Lifetime smoking	51 (23.3)	45 (21.6)	0.9 (0.4,2.2)	0.84	33 (15.6)	63 (29.2)	0.5 (0.2, 1.3)	0.16	*		0.15

*Note:* The groups were classified into two factors: (1) Islam (Islamic-based program), which comprises the Islamic and combined groups, (2) health (health-based program), which comprises the health and combined groups

^a^Main effects of Health intervention program using linear mixed effects model, adjusted for baseline scores

^b^Main effects of Islamic intervention program using linear mixed effects model, adjusted for baseline scores

^c^Interaction between health and Islamic program represents the additional effect of being in the combined group beyond the separate main effects presented for health and Islamic programs

^d^ *p* value for main effects from mixed effects model

^e^ *p* value for interaction between health x Islamic programs

^f^ICC = Intra-class correlations coefficients, from mixed effects random intercept model

*Interaction health x Islamic were not assessed because main effects of health and Islamic were non-significant

**Non-estimable

**Table 3:** Outcomes at 6 months follow-up and comparison between intervention and control groups (*n* = 427)

Outcome measures	Health (*n* = 109)	Islamic (*n* = 101)	Combined (*n* = 110)	Control (*n* = 107)	*p* – values
Health knowledge	11.3 ± 1.9***	11.7 ± 2.0***	10.9 ± 1.9***	8.9 ± 2.4	<0.001
Islamic knowledge	11.5 ± 2.5	14.5 ± 1.6***	12.0 ± 2.8	11.6 ± 2.2	<0.001
Smoking attitude	39.2 ± 7.9***	34.9 ± 7.2***	38.4 ± 6.6***	41.4 ± 8.7	<0.001
Smoking intention next year	1.3 ± 0.8***	1.0 ± 0.2***	1.3 ± 0.6**	1.5 ± 0.8	<0.001
Smoking intention in senior high school	1.5 ± 0.6*	1.0 ± 0.2***	1.4 ± 0.7***	1.6 ± 0.8	<0.001
Smoking intentions when older	1.5 ± 0.6	1.2 ± 0.5***	1.4 ± 0.7*	1.6 ± 0.8	<0.001
Past 7 days’ smoking behaviors	1.1 ± 0.4	1.0 ± 0.0	1.0 ± 0.0	1.1 ± 0.4	0.38
Past month’s smoking behaviors	1.1 ± 0.5	1.0 ± 0.0	1.0 ± 0.3	1.1 ± 0.4	0.14
Lifetime smoking behaviors	2.1 ± 2.2	1.2 ± 0.7*	1.4 ± 1.0	1.7 ± 1.3	0.01

*Note*: *p* values were obtained using ANCOVA tests, controlling for baseline. Total scores for health and Islamic knowledge ranged from 0 to 20, with higher scores indicating greater knowledge; attitude scores ranged from 0 to 100, with higher scores indicated more favorable attitudes towards smoking; smoking intentions scores ranged from 1 (“certainly not”) to 5 (“certain to smoke”); the past week smoking scores ranged from 1 (no cigarettes) to 6 (smoked six cigarettes or more); the past month’s smoking behavior scores ranged from 1 (none) to 7 (smoked more than 20 cigarettes per day), and; the lifetime smoking behavior scores ranged from 1 (never) to 9 (smoked more than 100 cigarettes)

*p < 0.05, **p < 0.01, ***p < 0.001, compared with control group. All values are presented as mean ± SD

### Program effects on Islamic knowledge at 6 months follow-up

Main effects and interaction effects of the health and Islamic-based intervention programs on Islamic knowledge at 6 months follow-up are shown in Table 2. Main effects of the interventions appeared to be significant for the Islamic (β = 2.2 ± 0.5, *p* < 0.001) and health-based intervention (β = 1.1 ± 0.4, *p* = 0.01) at 6 months follow-up compared to the control group. There was a significant interaction between health and Islam (β = −2.3 ± 0.9, *p* < 0.01), suggesting that the effects of the combined program on Islamic knowledge depended upon inclusion of the health-based intervention component and vice versa, i.e. the effects were significantly less than additive in the combined group. The effects were more homogenous for participants within the same class (ICC = 0.09). Groups’ differences in Islamic knowledge at 6 months follow-up are presented in Fig. 2 and Table 3. There were differences between the intervention and control groups in Islamic knowledge scores at 6 months, with the scores significantly higher in the Islamic-based program, but not in the health-based or combined programs, when compared to control group (Table 3).

### Program effects on smoking attitudes at 6 months follow-up

Main effects and interaction effects of the health and Islamic-based intervention programs on smoking attitudes at 6 months follow-up are presented in Table 2. Main effects of the interventions were significant for the health (β = −11.5 ± 1.8, *p* < 0.001) and Islamic-based intervention (β = −6.0 ± 1.9, *p* < 0.01) at 6 months follow-up. There was no significant interaction effects (β = 6.1 ± 3.2, *p* = 0.07) between the health and Islam at 6 months follow-up, suggesting that there were no differential effects on the reduction of smoking attitude scores for both the health and Islamic based interventions, i.e. the effects were additive in the combined group. The effects were more similar for participants within the same class (ICC = 0.07). Groups’ differences in smoking attitudes at six months are presented in Table 3 and Fig. 2. Table 3 shows there were significant differences (*p* < 0.001) between groups in smoking attitude at 6 months follow-up, with the three intervention groups having significantly lower scores in smoking attitude scores when compared to control groups.

### Program effects on intentions to smoke tobacco at 6 months follow-up

Main effects and interaction effects of health and Islamic-based intervention programs on intentions to smoke tobacco at 6 months follow-up are presented in Table 2. There was no significant main effects on intention to smoke tobacco next year (OR = 0.4, 95 % CI = 0.1–1.3, *p* = 0.14), during senior high school (OR = 0.4, 95 % CI = 0.1–1.3, *p* = 0.13), and when older (OR = 0.5, 95 % CI = 0.2–1.2, *p* = 0.13) for the health based intervention programs at 6 months follow-up. Main effects of the Islamic based intervention programs were not significant on intention to smoke tobacco next year (OR = 0.4, 95 % CI = 0.1–1.6, *p* = 0.20), during senior high school (OR = 0.3, 95 % CI = 0.1–1.2, *p* = 0.10), and when older (OR = 0.6, 95 % CI = 0.2–1.4, *p* = 0.24) at 6 months follow-up. The effects were more similar between participants within the same class for intention to smoke next year (ICC = 0.12), during senior high school (ICC = 0.12), and when older (ICC = 0.07). Groups’ differences in intentions to smoke tobacco at 6 months follow-up are presented in Table 3 and Fig. 2. There were significant differences (*p* < 0.001) between groups with respect to their intention to smoke tobacco next year, during senior high school and when older, with the intention to smoke significantly reduced in each of the three intervention groups when compared to the control group (Table 3).

### Program effects on smoking behaviors at 6 months follow-up

Table 2 demonstrates main effects and interaction effects between health and Islamic-based intervention programs on smoking behaviors at 6 months follow-up. There was a significant main effects on smoking behaviors in the past 30 days for the health- (OR = 0.4, 95 % CI = 0.2–1.0, *p* = 0.04) and Islamic-based interventions (OR = 0.1, 95 % CI = 0.0–0.8, *p* = 0.03) at 6 months follow-up but the effects were insignificant in the past week and lifetime smoking behaviors for both the health and Islamic-based interventions. The effects appeared to be more similar between participants within the same class for their smoking behaviors in the past week (ICC = 0.24), past 30 days (ICC = 0.10) and lifetime (ICC = 0.15). Groups’ differences in tobacco smoking behaviours at 6 months follow-up are presented in Table 3 and Fig. 2. Compared to control group, the frequency of tobacco smoking for the health and combined programs were not significantly different in the past week, past 30 days, and lifetime but differed significantly with those in the Islamic-based interventions for the lifetime smoking behaviors.

## Discussion

This study was intended to assess the impact of three types of school-based smoking prevention program approaches namely the health, Islamic and combined program amongst adolescents in the Aceh Province, Indonesia on their smoking knowledge, attitude, intentions, and behaviors at 6-month follow-up. Findings of this study provide evidence that school-based smoking prevention programs remained effective at a 6-month post-interventions time-point.

An important finding of this study is significantly higher scores on knowledge and those who had received Islamic- or health-based education components, these groups remained more knowledgeable than those in the comparison groups (non-health or non-Islam) at 6 months follow-up. After initial effects in increasing knowledge scores for participants in both the health and Islamic-based intervention programs over 1 week of the programs completion [12], sustained effects were observed at 6 months follow-up. This finding supports previous studies [25, 26] regarding the long-term effectiveness of school-based smoking programs on knowledge improvement. It must be noted, however, that the combined program was not effective in retaining an increase in participants’ Islamic knowledge at 6 months follow-up. Although students in the combined program had also been exposed with important concepts of the Islamic-based interventions material, their Islamic knowledge scores were non-significantly higher to those in the comparison group. It appears, therefore that a consistent program of education in Islam is required to achieve long-term program effectiveness for this outcome.

Moreover, the three intervention programs improved participants’ smoking attitudes at 6 months follow-up. The inclusion of the health and Islamic based intervention programs appeared to have a significant impact on individual’s smoking attitudes at 6 months after program completion. The reduction in mean smoking attitude scores by 9.9 points (*p* < 0.001) for the health and 7.2 points (*p* < 0.01) for the Islamic based intervention programs after 6 months intervention were larger than immediately after the interventions, where it was equivalent to shift of 5.8 points (*p* = 0.14) for the health and 5.3 points (*p* < 0.001) for the Islamic based intervention programs. Previous studies have provided mixed conclusions about program effects on smoking attitude. Although some studies [26–28] found significant longer term effects, others have not [25]. A religion-based intervention has also been reported to have had a positive impact on anti-smoking attitudes among students [29]. It is a general belief that religiosity/spirituality could provide positive effects on adolescents’ health attitudes [30]. Indeed, religion is perceived as an important aspect of people’s lives [31], behavioral and lifestyle choice [29].

Our study also offers evidence for significant effects of school-based programs in reducing smoking intentions in the long-term. An evaluation immediately after the end of the intervention programs showed some reductions in smoking intentions in next year, during senior high school, and when older in the health and Islamic based interventions [12]. Similar downward trends were observed in the separate health and Islamic based interventions at 6 months follow-up. The findings suggest that smoking intentions remained lower among participants in the three intervention programs than in the control group at 6 months after the program interventions completion. Although the combined health and Islamic based intervention program failed to show substantial a reduction in tobacco smoking intentions, there was evidence that the inclusion of Islamic-based components provided benefits. Similarly, a previous study [32] has indicated the effectiveness of a school-based program in reducing smoking intentions at 6 months follow-up. Additionally, it is important to note that the present study involved Muslim adolescents only. While tobacco smoking is considered as age-inappropriate [13] and religiously forbidden behaviors for minors by the Indonesian Ulema Council [33], the benefits of tailoring school-based program interventions with participant’s religious background was also evidenced in this study. In fact, cultural sensitivity is an important factor for program effectiveness [34].

Our study appears to support long-term effectiveness of school-based program on smoking behaviors. The proportion of tobacco smoking was marginally lower among participants in the three intervention programs than in control group. Effects of the Islamic-based program in reducing lifetime smoking behaviors were stronger than in the other programs. Since smoking rates at baseline were very low, the observed reductions in the assessed smoking behaviors in the health and Islamic-based programs were not detected as being statistically significant. Nevertheless, there was sufficient evidence to suggest that both the health and Islamic-based intervention programs provided almost similar effects on the reduction of lifetime smoking behaviors at 6 months follow-up. Findings from a systematic review about long-term effectiveness of school-based smoking prevention programs on smoking behaviors have been less promising. While Wiehe et al. [9] found that seven of eight studies in their review failed to decrease smoking behaviors among program participants in long term, Flay [11] suggests and others [26, 35] have found that school-based smoking prevention programs can be effective longer term.

There were several limitations of this study including the lack of biochemical validation for students’ response toward self-report questionnaires; the relatively short time evaluation (6 months) of program impact assessment [12] and; the inclusion of study participants from a relatively small geographical area. Data in the current study relied upon students’ responses (self-report, paper-based questionnaires) and the use biochemical tests would be useful to ensure the validity of students’ response toward questionnaire items regarding tobacco use. Although the validity and reliability of survey items had been verified and students had been informed about confidentiality of response surveys, any biases associated with the use of the self-report surveys might influence this study’s findings.

This study reported program impacts at 6-months follow-up and a longer-term assessment is required to determine when program effectiveness may diminish. There is still insufficient evidence about the longer-term effects of school-based smoking prevention programs at present, especially in Indonesia.

Participants in this study were recruited from the capital of the Aceh Province. Thus, its generalization to other areas may be limited. Smoking prevalence differs by geographic area and social economic status in Indonesia, and predominates among males in rural areas [36], individuals with low education level [37], and amongst poorer individuals [37]. Future study therefore should include students from a broader area, with a larger participants (more schools per arm) and with a variety of religious backgrounds. Such studies will broaden our understanding about the effectiveness of these programs, including the strengths and weaknesses of the programs when implemented in other populations. Although many students benefited from this study, many more could also benefit with an extended program. As has been stated [6] the proportion of tobacco users across the community levels is very high in Indonesia. Tobacco smoking has not only been regarded as a culturally accepted behavior but essential in the social and political lives of people in Indonesia [38], For many young Indonesians, tobacco smoking is viewed as part of identity and linked with social and cultural-religious practice [39]. Although some positive progress have been made in tobacco control and regulation in Indonesia including in tobacco advertisement recently, the efforts remain in adequate and failed to meet the WHO’s [5] recommendation for tobacco control measures. Given these consideration, the successfulness of this study could provide insight for policy makers and boost the existing efforts in smoking prevention and cessation program across the country, if any.

## Conclusions

This is the first study to observe longer term effects of school-based smoking prevention programs in Indonesia. Findings provide further support that school-based programs can provide longer term effects on the improvement of individual knowledge (Health-based program: β = 4.3 ± 0.4, *p* < 0.001 for health knowledge and β = 1.1 ± 0.4, *p* = 0.01 for Islamic knowledge. Islamic-based program: β = 3.7 ± 0.4, *p* < 0.001 for health knowledge and β = 2.2 ± 0.5, *p* < 0.001 for Islamic knowledge) and anti-smoking attitudes (Health-based program: β = −11.5 ± 1.8, *p* < 0.001. Islamic-based program: β = −6.0 ± 1.9, *p* < 0.01) and the reduction of smoking behaviors among adolescents (Islamic-based program: OR = 0.1, 95 % CI = 0.0 – 0.8, *p* = 0.03 for the past month smoking behaviors. Health-based program: OR = 0.1, 95 % CI = 0.0 – 0.8, *p* = 0.04 for the past month smoking behaviors). Health and Islamic-based programs provided stronger effects and while the separate health and Islamic-based approaches showed similar effects, Tailoring intervention components with participants’ religious background might be useful in improving the long-term effectiveness of school-based smoking prevention programs.
